# Reduction of Sulfur Compounds through Genetic Improvement of Native *Saccharomyces cerevisiae* Useful for Organic and Sulfite-Free Wine

**DOI:** 10.3390/foods9050658

**Published:** 2020-05-20

**Authors:** Alice Agarbati, Laura Canonico, Francesca Comitini, Maurizio Ciani

**Affiliations:** Department of Life and Environmental Sciences, Polytechnic University of Marche, 60131 Ancona, Italy; a.agarbati@pm.univpm.it (A.A.); f.comitini@univpm.it (F.C.)

**Keywords:** native *Saccharomyces cerevisiae*, organic wine, sulfite-free wine, hydrogen sulfide, sulfur dioxide

## Abstract

Sulfites and sulfides are produced by yeasts in different amounts depending on different factors, including growth medium and specific strain variability. In natural must, some strains can produce an excess of sulfur compounds that confer unpleasant smells, inhibit malolactic fermentation and lead to health concerns for consumers. In organic wines and in sulfite-free wines the necessity to limit or avoid the presence of sulfide and sulfite requires the use of selected yeast strains that are low producers of sulfur compounds, with good fermentative and aromatic aptitudes. In the present study, exploiting the sexual mass-mating spores’ recombination of a native *Saccharomyces cerevisiae* strain previously isolated from grape, three new *S. cerevisiae* strains were selected. They were characterized by low sulfide and sulfite production and favorable aromatic imprinting. This approach, that occurs spontaneously also in nature, allowed us to obtain new native *S. cerevisiae* strains with desired characteristics that could be proposed as new starters for organic and sulfite-free wine production, able to control sulfur compound production and to valorize specific wine types.

## 1. Introduction

*Saccharomyces cerevisiae* is the yeast species mainly used as starter culture in winemaking for its peculiar physiological and biotechnological properties [1]. *S. cerevisiae* is characterized by a vigorous alcoholic fermentation under both aerobic and anaerobic conditions [2,3] showing high competitiveness toward other yeasts that commonly colonize fresh grape juice. On the other hand, their wide use as starter of fermentation, can lead to the standardization of the analytical and sensory properties of wines [4,5]. In this regard, the use of selected native *S. cerevisiae* strains as starters of fermentation could be a suitable strategy to overcome the production of standardized wines.

Indeed, native yeasts represent a great reservoir of biodiversity characterized by peculiar properties that could be exploited in winemaking to improve the aromatic profile of wines [6,7,8,9,10,11]. Several studies have highlighted the genotypic and phenotypic differences among native *S. cerevisiae* yeasts, and also in relationship with the geographical distribution of the isolated strains [12,13,14,15,16,17,18]. However, the isolation of native *S. cerevisiae* yeasts with all desired oenological features is difficult to find in environmental niches.

In addition, these native yeasts could overproduce some undesirable metabolites such as acetic acid or sulfur compounds that lead to sensory defects [19,20,21]. For these reasons, different strategies could be used to generate new yeast strains with tailored characteristics. For example, De Vero and co-workers [22], through sexual recombination of spores and specific selection pressure, generated *S. cerevisiae* strains characterized by low sulfite and impaired H_2_S production. Additionally, Liu and co-workers [23] used molecular approaches to increase yeast biodiversity by obtaining novel yeasts with optimized fermentation performance and improved wine quality.

Sulfur compounds in wine represent one of the most important parameters determining the acceptability for the wine marketing [24]. In particular, sulfites (SO_2_) and sulfide (H_2_S) are naturally produced by yeasts during sulfur assimilation pathways and an excessive production could confer negative rotten egg aroma, inhibit malolactic fermentation and represent a source of health concerns [25,26,27]. Sulfur dioxide is mainly used as an antiseptic agent against yeast and bacteria as well as an antioxidant agent. However, the toxicity and the allergenic potential of this additive, together with more aware consumers, has given rise to new biotechnological approaches that have led to a significant reduction of sulfites in wines, as well as new kinds of wines such as organic and sulfite-free wines. The definition of “organic wine” is difficult because its laws and regulations differ worldwide. In Europe, it has been regulated by law since 2012 (EC Regulation No. 203/2012) [28]. For these reasons, the goal of the present study was to obtain new *S. cerevisiae* strains derived from the improvement of a native *S. cerevisiae* strain, previously isolated from Verdicchio grape variety and characterized by medium-high sulfur compounds production. Exploiting the sexual recombination of its spores, it was possible to obtain three new *S. cerevisiae* strains characterized by a low production of sulfur compounds and a peculiar aromatic imprinting. These new *S. cerevisiae* strains were then tested for their fermentation performance.

## 2. Materials and Methods

### 2.1. Native S. cerevisiae Strain

The native *S. cerevisiae* strain DiSVA 705 used in this study was obtained from the yeasts collection of Department of Life and Environmental Sciences (DiSVA) of Polytechnic University of Marche (Ancona, Italy). It was previously isolated from Verdicchio grape variety and chosen for its oenological properties. It was also characterized by medium-high sulfur compounds production. The yeast was maintained at 4 °C for short-term storage in YPD agar medium (1% yeast extract, 2% peptone, 2% dextrose, 2% agar) (Oxoid, Basingstoke, UK) and for long-term storage in YPD broth supplemented with 80% (*w*/*v*) glycerol, at −80 °C.

### 2.2. Yeast’s Sporulation Procedure and Spore Analyses for H_2_S Production 

The native strain DiSVA 705 was cultivated in YPD broth for 24 h at 25 °C in a rotary shaker (200 rpm), then 20 µL of the cell suspension was spread on Sporulation Medium (0.25% yeast extract, 0.1% glucose, 0.98% potassium acetate, 2% agar) [29] and incubated at 23 °C for at least 5 days. When tetrads were observed, a spot of the culture was resuspended in 45 µL of sterile distilled water containing 5 µl of Zymolyase 100-T (ICN Biomedicals, Inc., Irvine, CA, USA) solution (4 mg/mL of sorbitol 2 M) and incubated at room temperature for 10 min to facilitate the cell wall disruption. Ten tetrads were dissected using a micromanipulator (Singer SMS Manual, Somerset, UK) and the single spores were transferred to new YPD-agar plates and grown at 25 °C for 48 h. The viable spores were then analyzed for their H_2_S production, spreading them on BiGGY agar medium (Oxoid Ltd., Cheshire, England) and incubated at 25 °C for 48 h. In this medium, the colonies appear white for those H_2_S-negative and brown-black for those H_2_S-positive.

### 2.3. Sexual Recombination of Spores and New Strain Selection

The tetrads obtained through the sporulation procedure were suspended in the Zymolyase solution used previously, mixed briefly and incubated at room temperature for 30 min to allow the restoration of diploid state and the genetic rearrangement by the random conjugation of gametes. Subsequently, 10 µL of the suspension was spread on YPD agar medium and incubated at 25 °C for 24 h. About 100 colonies were randomly selected as potential new strains and analyzed for their H_2_S production and mitochondrial activity using GLY medium (2% peptone, 1% yeast extract, 3% glycerol, 1% ethanol (added after autoclaving), 2% agar) [29] containing non-fermentable glycerol as unique carbon source. Out of 100 colonies, 4 were chosen as potential new *S. cerevisiae* strains (G4, I1, I4 and B4) and used in the next steps of the investigation.

### 2.4. Molecular Fingerprinting

The selected colonies (H_2_S^−^) were submitted to molecular fingerprinting comparing their electrophoretic profile with those of the native strain DiSVA 705, to verify the genetic rearrangement. The whole genome DNA of each strain analyzed was extracted as follow: 700 µL of YPD broth has been inoculated with each colony separately and let it grow overnight at 30 °C in shaker (200 rpm). The overnight cultures were centrifuged 3 min at 3000 rpm and the supernatant removed. The cell pellets were resuspended in 200 µL of TE-buffer (10 mM Tris-HCl, 1 mM EDTA, pH 8.0), 250 µL of glass beads (0.45 mm diameter) and 200 µL of PCI (25:24:1, phenol pH 8:chlorophorm:isoamyl alcohol). The cells were lysed for 2:30 min at 30 Hz (twice) and centrifuged 20 min at 3000 rpm, 4 °C to spin down cellular detritus. The upper aqueous phase (DNA) (c.a, 100–150 µL) was collected in a new tube and 800 µL of diethylether were added. After 20 s at 30 Hz of vortex, the tubes were centrifuged 20 min at 3000 rpm, 4 °C and the diethylether completely removed leaving the tubes uncapped under laminar flow hood. The quality and concentration of DNA were checked by Nanodrop (ND-8000, 8-Sample Spectrophotometer, Thermo Fisher Scientific, Waltham, MA, USA) and the DNA obtained was conserved at −20 °C. Molecular characterization of interdelta sequences was performed using two primer pairs: δ 2/12 and δ 12/21 as described by Legras and Karst [30]. PCR products (δ 12/21) were separated by automated capillary electrophoresis QIAxcel Advanced system (Qiagen, Venlo, The Netherlands) with a Screening Gel Cartridge (Qiagen, Venlo, The Netherlands) under the following parameters: sample injection voltage 5 kV, sample injection time 10 s, separation voltage 5 kV and separation time 420 s. The QX Size Marker 50 bp/5kb (Qiagen, Venlo, The Netherlands) was used for fragment size and the QX Alignment Marker for 50 bp/5kb (Qiagen, Venlo, The Netherlands) was used to align the resulting fragments.

### 2.5. Genomic Stabilization

The genomic stabilization of the new strains was necessary to ensure their genetic stability over time. Each strain was precultured in modified YPD broth (0.5% yeast extract, 0.1% peptone, 5% glucose) for 24 h at 25 °C under stirrer condition (200 rpm); then 7.5 µL of preculture was used to inoculate 750 µL of synthetic grape juice (SGJ) [31]. The initial yeast concentration was measured spectrophotometrically (OD_600_) and each culture left to grow up at 25 °C for 1 week. Then, the yeast cultures were transferred in fresh SGJ with following the same procedure described above, OD_600_ measured and left to grow up at the same conditions. This procedure was repeated for three weeks, until to reach approximately 20 yeast generations (c.a 7 generations per week), following the procedure described by Steensels et al. [32]. To check the homogeneity of the population, 7 colonies of each of the stabilized cultures were subjected to genetic fingerprinting using primer pair δ 12/21 as previously described, to compare the genotype before and after stabilization. The native strain DiSVA 705 was subjected to the same procedure as control. All the colonies were also tested for their H_2_S production, as previously described.

### 2.6. Fermentative Aptitudes: Microvinification Trials

The fermentative aptitudes of the improved stabilized strains were tested in both SGJ [31] and natural grape juice (NGJ) (organic Verdicchio grape juice). The Verdicchio must that was used, coming from a 2015 vintage, had the following analytical composition: initial sugar content 217 g/L, total SO_2_ 25 mg/L; malic acid 2.8 g/L, total acidity 4.53 g/L, pH 3.26 and nitrogen content YAN 111 mg/L. The low SO_2_ content in organic NGJ is due to a limited use of potassium metabisulfite on grapes at harvest time. All the strains were precultured in modified YPD broth for 24 h at 25 °C under stirrer condition (200 rpm) and then used to inoculate flasks containing SGJ (150 mL) and NGJ (70 mL) with an initial yeast concentration of 1 × 10^6^ cells/mL; both trials were conducted in triplicate. All the flasks were locked with a Müller valve. The flasks containing SGJ and NGJ were placed at 20 °C ± 2 °C, under static condition. The results were compared with the native strain DiSVA 705 and with the commercial strain Lalvin ICV OKAY (Lallemand Inc., Toulouse, France) used in winemaking and characterized for the absence of H_2_S production. The fermentation kinetics of the yeasts were expressed as the weight loss of the flasks (due to the CO_2_ evolution) monitored from the beginning to the end of the fermentations (i.e., constant weighing for 3 consecutive days).

#### 2.6.1. Main Analytical Compounds of Wines

The H_2_S production was evaluated during the fermentation by acetate strips (CARLO ERBA Reagents S.r.l., Milan, Italy). In SGJ trials the ethanol content was measured by DMA 4500 M density meter and Alcolyzer Beer ME (Anton Paar, Graz, Austria), while acetic acid and SO_2_ were quantified using Gallery™ Plus Beermaster (ThermoFisher, Vantaa, Finland) discrete photometric analyzer. In NGJ trials ethanol, total acidity, acetic acid and SO_2_ were determined following the indications of Canonico et al. [33]. The sugar content, malic acid and ammonium were determined using specific enzyme kits (Megazyme International Ireland). Free α-amino acids were determined using the o-phthaldialdehyde/N-acetyl-l-cysteine spectrophotometric assay. Yeast assimilable nitrogen was calculated as the sum of the concentrations of free α-amino acids and ammonium.

#### 2.6.2. Main Byproducts of Fermentation and Volatile Compounds

Acetaldehyde, ethyl acetate, n-propanol, isobutanol, amyl-and isoamyl alcohol were evaluated in SGJ and NGJ wines, while the main volatile compounds were detected only in NGJ (Verdicchio wines). Acetaldehyde, ethyl acetate, n-propanol, isobutanol, amyl and isoamyl alcohols were quantified by direct injection into a gas-liquid chromatography system (GC-2014; Shimadzu, Kjoto, Japan). Each sample was prepared and analyzed following the procedures of Canonico et al. [33]. The main volatile compounds were determined by solid-phase microextraction (HS-SPME) as reported by Canonico et al. [34].

### 2.7. Statistical Analyses

The means of the analytical compounds were compared via one-way ANOVA using STATISTICA 7 software (Statsoft, Tulsa, OK, USA) were used to the means analyses. The significant differences were obtained considering the associated *p*-value < 0.05 by Duncan test. Additionally, mean values of main byproducts of fermentation and volatile compounds were analyzed by Principal Component Analysis (PCA), carried out using JMP 11^®^ statistical software (Statistical discovery from SAS, New York, NY, USA). The mean data were normalized to eliminate the influence of hidden factors.

## 3. Results

### 3.1. H_2_S Production by the Viable Spores

**Figure 1:**
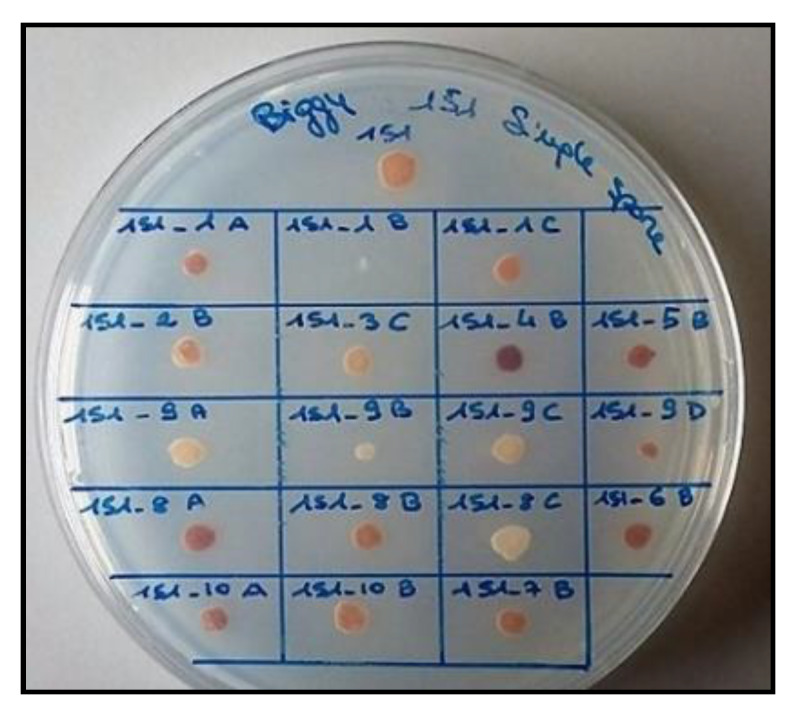
Different level of H_2_S production of the spores. The different color intensity of the colonies indicates different levels of H_2_S production. White colonies were H_2_S^−^ while brown/black colonies expressed maximum level of H_2_S. An intermediate color intensity indicated medium production of H_2_S.

Within ten selected tetrads, the spores’ mortality was 62.5%. The resulting viable ones were evaluated for the H_2_S production (Table 1, Figure 1). The results showed a great variability within the same tetrad. For example, as observed for the eighth tetrad: “A” spore showed the maximum expression of hydrogen sulfide, an intermediate production of this compound was detected for “B” spore, while completely absent was found for “C” spore.

### 3.2. Phenotypic Selection of the Improved S. cerevisiae Strains

Based on the production levels of H_2_S (white colonies) and respiratory efficiency (some strains defect in the respiratory chain, “petit mutants”) potential new strains were selected. G4, I1, I4 and B4 exhibited the desired phenotypic characteristics: H_2_S^−^ (unlike DiSVA 705 strain) and respiratory activity^+^ (like DiSVA 705 strain) excluding eventual “petite mutant” strains.

### 3.3. Molecular Fingerprinting of the Potential Improved Strains

**Figure 2:**
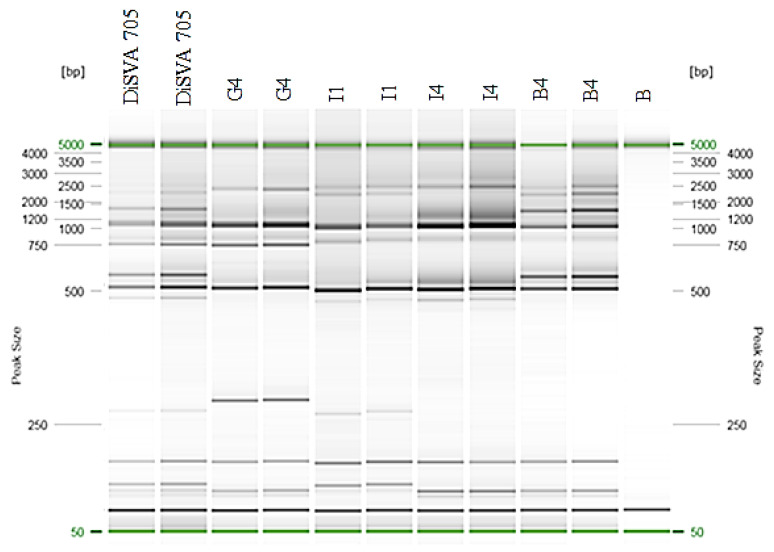
Molecular fingerprinting of the potential improved strains using primer pair δ 12–21. The native DiSVA 705 strain was used as control. Each strain was analyzed in duplicate to confirm. The QX Size Marker 50 bp/5 kb (Qiagen) was used for fragment size. Lane B was indicated as negative control.

Molecular fingerprinting of the selected G4, I1, I4 and B4 strains was carried out using interdelta primers (δ 12–21) and the results are reported in Figure 2. The electrophoretic gel showed a unique profile for the four improved strains in comparison with the native DiSVA 705 strain and each other. It was also confirmed by δ 2–12 profile.

### 3.4. Effects of the Genomic Stabilization on Interdelta Sequences and H_2_S Production of the Improved Strains

**Figure 3:**
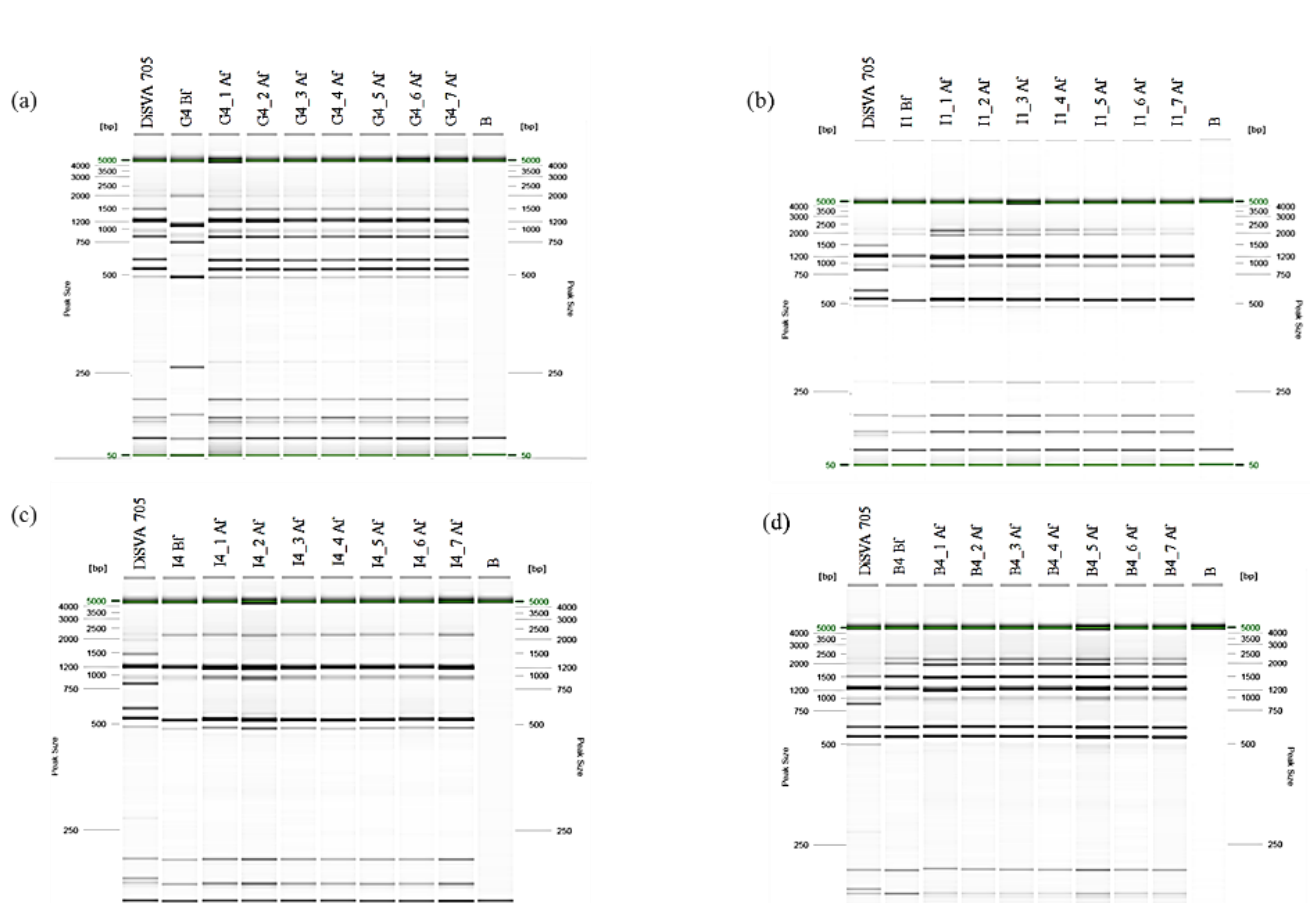
Electrophoretic profiles of interdelta sequences obtained by primer pair δ 12–21. Each stable isolate, belonging to each group, was named from 1 to 7 preceded by strain’s name and compared with the same unstabilized strain. The DiSVA 705 strain was used as control. The acronyms “Bf” and “Af” were used to indicate before and after genome stabilization, respectively. Lane B: indicated as negative control. The QX Size Marker 50 bp/ 5kb (Qiagen) was used for fragment size. (**a**–**d**) represent the electrophoretic profiles of G4, I1, I4 and I4, before and after stabilization, respectively.

The genomic stabilization of the four improved *S. cerevisiae* strains was carried out using SGJ medium and the DiSVA 705 strain was used as control. After stabilization, 7 colonies of each improved strain were isolated and subjected to molecular fingerprinting to check the population homogeneity and genome stability. The electrophoretic profiles of the stable colonies were compared with the control and with the profiles obtained before the stabilization procedure. Within the colonies belonging to the same improved and stable strain, all of them exhibited the same interdelta profile (Figure 3a–d), highlighting a sure population homogeneity within the stable G4, I1, I4 and B4. Additionally, comparing the profile of each potential new strain before and after stabilization, only G4 strain showed a different profile indicating its genomic instability; in particular, the stabilized G4 showed the same profile of DiSVA 705 (Figure 3a). Regarding the H_2_S production, all stabilized colonies maintained the characteristic observed before the stabilization (no or low H_2_S production) with the exception for G4 stable isolates that confirmed a medium/high H_2_S phenotype, similar to that exhibited by DiSVA 705 strain. These data revealed three improved strains obtained such as I1, I4 and B4.

### 3.5. Microvinification Trials

#### 3.5.1. Fermentation Kinetics

**Figure 4:**
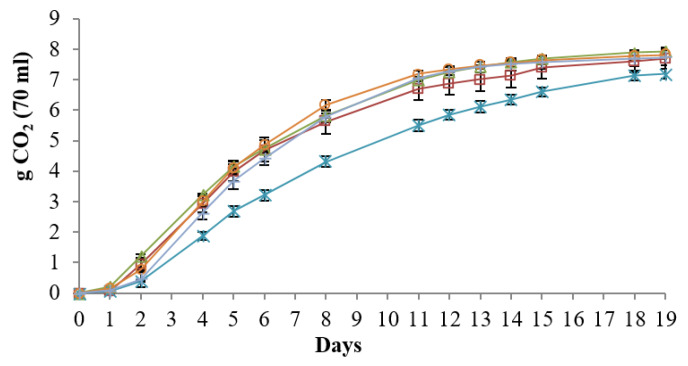
Fermentation kinetics of the new selected strains tested: OKAY (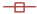); DiSVA 705 (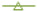); I1 (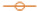); I4 (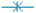); B4 (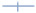). The graph represented the fermentation trend in NGJ. The results were the mean values and the standard deviations were represented as error bars.

The fermentation kinetics of the three improved trains (I1, I4, B4) detected during the fermentation of the NGJ were reported in Figure 4. The fermentation behavior of I1 and B4 showed a comparable behavior among them and with the controls (native *S. cerevisiae* DiSVA 705 and the OKAY commercial strain). Only I4 exhibited a slower kinetic than the other strains. Overall, the strains have reached the end of the fermentation after 19 days. The same trend was observed for the trials conducted in SGJ (data not shown).

#### 3.5.2. Main Analytical Compounds

The main analytical compounds of the final wines are shown in Table 2. The wines from SGJ exhibited ethanol contents comparable among the strains tested with the exception for I4 strain that showed the lowest ethanol production. This is in accordance with its lowest CO_2_ evolution during the fermentation. The same trend of I4 strain was observed in wines from NGJ. Acetic acid production was comparable between the strains and a reduction of the content of this compound was observed when NGJ was used. Additionally, variability in SO_2_ production was detected. Low levels of SO_2_ (<6 mg/L) were produced by yeasts when SGJ was used, while higher amounts were detected using NGJ. In particular, the commercial strain OKAY showed the lowest amount, while I1 and I4 produced intermediate amounts of SO_2_, lower than that exhibited by the parental strain DiSVA 705 (32.29 ± 0.87 mg/L). The H_2_S production was evaluated by acetate strips during both fermentations and no production of this compound was detected in all the trials, with the exception for DiSVA 705, by BiGGY agar plate assay.

#### 3.5.3. The Main Byproducts and Volatile Compounds of Fermentation

The main byproducts of fermentation such as acetaldehyde, ethyl acetate, n-propanol, isobutanol, amyl-and isoamyl alcohol were detected in SGJ wines (Table 3), while in NGJ key volatile compounds were also detected in addition to the main fermentation byproducts (Table 4). The results of wines from SGJ showed that all the yeasts produced acetaldehyde around 15 mg/L, only I1 strain produced higher level of this compound. Furthermore, the native and all improved strains exhibited a significant lower n-propanol production (c.a. 20 mg/L) than the commercial strain. The improved strains (I1, I4, B4) produced the lowest amyl-and isoamyl alcohols in comparison with the parental strain DiSVA 705 and OKAY (significant different), while regarding ethyl acetate and isobutanol production, variability among the strains was observed. NGJ wines showed a general higher production of byproducts. In particular, all non-commercial strains exhibited higher acetaldehyde and n-propanol content (with the exception of DiSVA 705 for n-propanol). Variability among the strains for ethyl acetate, isobutanol, amyl- and isoamyl-alcohol production was observed. Regarding the main volatile compounds evaluated in NGJ wines, OKAY exhibited the highest production of ethyl butyrate, phenyl ethyl acetate and β-phenyl ethanol, while the four native strains (DiSVA 705, I1, I4, B4) showed higher ethyl hexanoate production. Differently, I1, I4 and B4 showed higher isoamyl acetate production than DiSVA 705 and similar to wines fermented by OKAY.

#### 3.5.4. Principal Component Analysis (PCA)

**Figure 5:**
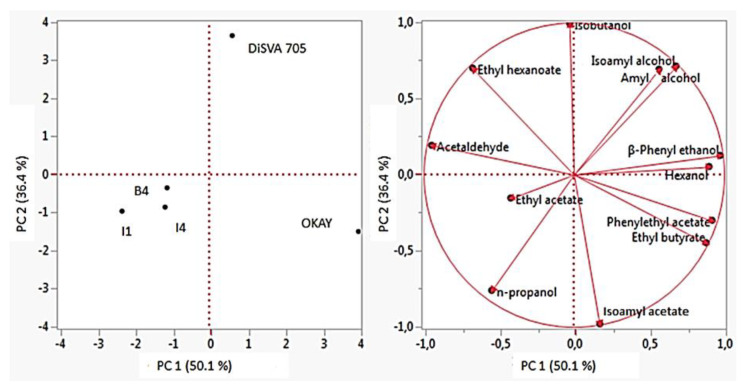
Principal component analysis based on the data for the main byproducts of fermentation and volatile compounds in the wines obtained NGJ.

PCA carried out on the data of the main fermentation byproducts and volatile compounds of the wines obtained from NGJ, was reported in Figure 5. The total variance explained was 86.40% (PCA1 = 50.1%; PCA 2 = 36.40%). The distribution of wines fermented by different strains showed that OKAY was into the lower right quadrant, DiSVA 705 strain was located in the upper right quadrant, while the three new strains I1, I4 and B4 were grouped into the lower left quadrant. The overall data mainly distinguished the new strains from both the OKAY and DiSVA 705 strains, through PCA1 (confirming the results of molecular fingerprinting). In particular, OKAY differentiated from the other strains for phenylethyl acetate and ethyl butyrate production, DiSVA 705 differentiated for isobutanol, amyl- and isoamyl-alcohol, while I1, I4 and B4 for acetaldehyde and n-propanol, ethyl hexanoate and isoamyl acetate production. The outcome of the PCA analysis showed clear differences among the strains tested regarding the aromatic compounds production during the fermentation and this reflects the ability of each strains to give a specific aromatic imprint to the wine.

## 4. Discussion

Metabolites released by yeasts during alcoholic fermentation influence aromatic and sensorial profile of wines [35,36]. Compounds derived from yeast sulfur metabolism, such as sulfites (SO_2_) and sulfide (H_2_S), may play a negative role in wine aromatic complexity, inhibit the malolactic fermentation and are negatively involved in health concerns, all these aspects suggest to limit or avoid their production by yeasts [21,27]. The production of organic wines and sulfite-free wines involve starter strains characterized by the absence or the reduced production of sulfur compounds. In these wines, the absence of SO_2_ requires a more severe limitation of contact with oxygen. In this strictly reduced environment, the negative effect of H_2_S on the aromatic profile of wines increases.

The production of sulfur compounds in wine is strictly linked to the yeast metabolism of sulfur-containing amino acids such as cysteine and methionine. The selection of new *S. cerevisiae* strains able to produce low concentrations of these undesirable compounds is one of the main goals of researchers to satisfy winemakers and consumer requests.

In the present work, a native *S. cerevisiae* strain [37], previously selected for its enological aptitudes, was subjected to mass-mating of its spores. This procedure allowed us to obtain three improved strains of *S. cerevisiae* (I1, I4, B4) that are low producers of sulfur compounds and able to provide a specific aromatic imprint to wines. This approach was used in previous works that exploited the sexual recombination of spores and specific selective pressure to generate non-genetically modified *S. cerevisiae* with desired oenological characteristics [22,38].

The gene expression variability in the three improved strains I1, I4 and B4 could be due to the presence of different gene alleles which randomly segregated during the sporulation of the native strain, originating different allelic combinations. This hypothesis was confirmed by the spores’ analysis of the native DiSVA 705 strain, which showed a great variability for the H_2_S production. The improved strains selected for their desired characteristics were confirmed to be really new strains through interdelta-sequence profile analysis. Furthermore, the fermentation behavior of these improved strains was comparable with the commercial control strain with a reduction of SO_2_ production in comparison to the native strain (DiSVA 705). The results of microfermentation trials indicated that a general reduction in acetic acid production and an increase of SO_2_ and by products (particularly higher alcohols) in NGJ fermentation trials were found. These differences were probably due to a more complex and complete composition of NGJ (i.e., solid particles, nitrogen composition, initial SO_2_ content) [39,40]. On the other hand, the improved strains, in addition to the lower production of sulfur compounds (H_2_S and SO_2_), released in wine a mix of fermentation byproducts such as ethyl hexanoate, isoamyl acetate and β-phenyl ethanol responsible of fruity and floral aroma to the final wine [8]. This behavior led the aromatic complexity and desirable footprint for Verdicchio wine (the same grape variety from which the native DiSVA 705 was isolated). Furthermore, the influence of the yeasts on the final wine quality was strictly strain dependent, as described also by Torrens et al. [41] Concerning the three improved strains, even if showed a different aromatic profiles, they coming from the same genetic heritage of native *S. cerevisiae* strain isolated from Verdicchio grape variety. Indeed, the aromatic profile of improved strains should come from the native strain even if with some differences. This fermentation behavior could improve the uniqueness of organic and sulfite-free wines produced specifically in Verdicchio wine area. However, this feature needs to be further investigated. In this regard, several studies described the important role of native yeasts to confer a specific aromatic imprinting to the wine recognizable with the territory of production, as a biogeography signature of the yeasts used [8,42,43,44]. The latter aspect represents a valorization to their use as new fermentative starter strains in organic wine production.

## 5. Conclusions

In conclusion, the use of mass-mating spores—a strategy that occurs spontaneously in nature between yeasts—improved native *S. cerevisiae* strains, as the reduced production of sulfite and sulfide was obtained. The analytical profile of wines indicated that they can confer to the wine a specific aromatic imprinting and could be used as new starter strains tailored for organic and sulfite-free wines. Further studies will be carried out to evaluate at the industrial level the potential use of these new naturally improved strains.

## Figures and Tables

**Table 1:** H_2_S production of the viable spores.

Spores Name	H_2_S Production
1_A	++
1_B	−
1_C	++
2_B	++
3_C	++
4_B	+++
5_B	+++
6_B	+++
7_B	++
8_A	+++
8_B	++
8_C	−
9_A	+
9_B	−
9_C	−
9_D	++
10_A	+++
10_B	++

H_2_S production of the viable spores. Each spore within the tetrad was named from “A” to “D” preceded by tetrad’s number. The symbols “+++”,“++”,“+” and “−” were used to indicate maximum (dark brown/black colony), medium (light brown colony), low (beige colony) and no (white colony) H_2_S production, respectively. It is well observable in the photo at the end of the table.

**Table 2:** The main analytical compounds of wines obtained from *S. cerevisiae* strains of this study.

	SGJ			NGJ		
Strains	Ethanol (% *v/v*)	Acetic Acid (g/L)	Total SO_2_ (mg/L)	Ethanol (% *v/v*)	Acetic Acid (g/L)	Total SO_2_ (mg/L)
OKAY	13.00 ± 0.15 ^ab^	0.81 ± 0.05 ^a^	0.29 ± 0.01 ^c^	13.65 ± 0.06 ^ab^	0.39 ± 0.02 ^b^	6.85 ± 0.49 ^e^
DiSVA 705	13.14 ± 0.02 ^a^	0.77 ± 0.01 ^ab^	3.81 ± 0.43 ^ab^	14.04 ± 0.17 ^a^	0.31 ± 0.02 ^c^	32.29 ± 0.87 ^a^
G4	13.17 ± 0.03 ^a^	0.74 ± 0.03 ^ab^	4.89 ± 2.06 ^a^	13.49 ± 0.24 ^b^	0.43 ± 0.01 ^b^	12.56 ± 0.45 ^d^
I1	13.15 ± 0.12 ^a^	0.72 ± 0.09 ^b^	5.40 ± 1.28 ^a^	13.84 ± 0.26 ^ab^	0.53 ± 0.03 ^a^	22.35 ± 0.78 ^b^
I4	12.71 ± 0.45 ^b^	0.73 ± 0.02 ^ab^	2.28 ± 1.43 ^bc^	12.74 ± 0.28 ^c^	0.49 ± 0.01 ^a^	17.44 ± 0.57 ^c^
B4	13.21 ± 0.01 ^a^	0.75 ± 0.00 ^ab^	0.76 ± 0.40 ^c^	13.69 ± 0.48 ^ab^	0.50 ± 0.05 ^a^	32.37 ± 1.05 ^a^

Data regarding synthetic grape juice (SGJ) and natural grape juice (NGJ) were reported at the left and right of the table, respectively. Data are means ± standard deviations and those with different superscript letters (^a–e^) within each column are significant (Duncan test; *p* < 0.05).

**Table 3:** Main fermentation byproducts in SGJ.

Main by-Products in SGJ (mg/L)	Yeast Strains				
Carbonyl Compounds	OKAY	DiSVA 705	I1	I4	B4
Acetaldehyde	15.21 ± 0.16 ^b^	13.91 ± 0.05 ^b^	17.96 ± 1.74 ^a^	13.84 ± 0.13 ^b^	15.12 ± 1.19 ^b^
**Esters**					
Ethyl acetate	18.69 ± 0.64 ^b^	22.29 ± 0.31 ^a^	17.54 ± 0.26 ^b^	11.27 ± 2.08 ^d^	14.50 ± 0.28 ^c^
**Alcohols**					
n-propanol	41.61 ± 0.44 ^a^	19.24 ± 0.41 ^b^	20.53 ± 0.51 ^b^	21.99 ± 4.87 ^b^	19.02 ± 0.54 ^b^
Isobutanol	19.66 ± 0.27 ^a^	14.81 ± 0.14 ^b^	7.69 ± 0.85 ^c^	6.61 ± 0.50 ^d^	8.61 ± 0.32 ^c^
Amyl alcohol	18.88 ± 0.44 ^a^	12.24 ± 0.17 ^b^	8.88 ± 0.82 ^c^	9.08 ± 0.64 ^c^	8.11 ± 1.10 ^c^
Isoamyl alcohol	47.51 ± 0.11 ^a^	35.90 ± 0.05 ^b^	29.41 ± 1.22 ^c^	29.45 ± 2.60 ^c^	27.35 ± 2.95 ^c^

Data are means ± standard deviations and those with different superscript letters (^a–d^) within each row are significant (Duncan test; *p* < 0.05).

**Table 4:** Main fermentation byproducts and volatile compounds in NGJ.

Main by-Products in NGJ (mg/L)	Yeast Strains				
Carbonyl Compounds	OKAY	DiSVA 705	I1	I4	B4
Acetaldehyde	12.53 ± 0.05 ^e^	63.46 ± 0.47 ^c^	89.66 ± 0.10 ^a^	70.52 ± 0.02 ^b^	57.00 ± 0.01 ^d^
**Esters**					
Isoamyl acetate	1.53 ± 0.21 ^a^	0.37 ± 0.08 ^c^	1.26 ± 0.15 ^ab^	1.27 ± 0.01 ^ab^	1.05 ± 0.16 ^b^
Phenylethyl acetate	0.77 ± 0.00 ^a^	0.14 ± 0.05 ^b^	0.10 ± 0.05 ^bc^	0.04 ± 0.01 ^c^	0.05 ± 0.01 ^c^
Ethyl hexanoate	0.08 ± 0.01 ^d^	0.34 ± 0.01 ^a^	0.26 ± 0.01 ^bc^	0.22 ± 0.00 ^c^	0.30 ± 0.06 ^ab^
Ethyl butyrate	0.87 ± 0.09 ^a^	0.03 ± 0.01 ^b^	0.09 ± 0.00 ^b^	0.07 ± 0.00 ^b^	0.06 ± 0.01 ^b^
Ethyl acetate	20.38 ± 0.57 ^b^	20.58 ± 1.38 ^b^	31.15 ± 0.11 ^a^	16.67 ± 0.89 ^c^	22.09 ± 0.65 ^b^
**Alcohols**					
n-propanol	40.56 ± 1.27 ^d^	20.95 ± 0.39 ^e^	59.30 ± 0.34 ^b^	69.83 ± 1.00 ^a^	49.36 ± 0.17 ^c^
Isobutanol	5.65 ± 0.42 ^c^	17.18 ± 0.53 ^a^	7.34 ± 0.40 ^b^	7.77 ± 0.76 ^b^	7.60 ± 0.16 ^b^
Amyl alcohol	14.60 ± 1.53 ^b^	19.76 ± 0.98 ^a^	8.70 ± 0.13 ^d^	12.20 ± 0.92 ^c^	6.85 ± 0.42 ^d^
β-Phenyl ethanol	24.93 ± 1.32 ^a^	16.24 ± 0.75 ^b^	8.54 ± 0.82 ^c^	6.28 ± 0.33 ^cd^	5.81 ± 1.07 ^d^
Hexanol	0.09 ± 0.02 ^a^	0.07 ± 0.00 ^ab^	0.05 ± 0.01 ^b^	0.06 ± 0.01 ^b^	0.07 ± 0.01 ^ab^
Isoamyl alcohol	94.21 ± 1.25 ^b^	115.61 ± 0.70 ^a^	58.03 ± 0.29 ^d^	56.51 ± 0.36 ^d^	62.35 ± 0.36 ^c^

Data are means ± standard deviations and those with different superscript letters (^a–e^) within each row are significant (Duncan test; *p* < 0.05).
